# Delayed Recognition: A Co-Citation Perspective

**DOI:** 10.3389/frma.2020.577131

**Published:** 2021-02-19

**Authors:** Wenxi Zhao, Dmitriy Korobskiy, George Chacko

**Affiliations:** ^1^ Netelabs, NET ESolutions (an NTT DATA Company), McLean, VA, United States; ^2^ Grainger College of Engineering, University of Illinois Urbana-Champaign, Urbana, IL, United States; ^3^ Department of Computer Science, University of Illinois Urbana-Champaign, Urbana, IL, United States

**Keywords:** delayed recognition, co-citation, bibliometrics, graph database, sleeping beauty

## Abstract

A Sleeping Beauty is a publication that is apparently unrecognized by citation for some period of time before experiencing a burst of recognition. Various reasons, including resistance to new ideas, have been attributed to such delayed recognition. We study this phenomenon in the special case of co-citations, which represent new ideas generated through the combination of existing ones. Using relatively stringent selection criteria derived from the work of others, we analyze a very large dataset of over 940 million unique co-cited article pairs, and identify 1,196 cases of delayed co-citations. We further classify these 1,196 cases with respect to amplitude, rate of citation, and disciplinary origin.

## 1. Introduction

The term “Sleeping Beauty” has been used to describe an article that is not well cited in the early years after its publication but experiences a sharp increase in the rate at which it is subsequently cited (van Raan, 2004). An implication is that the concept presented in such an article is “ahead of its time”, and that 93 resistance to its ideas may have delayed its recognition.

Causes for resistance (Barber, 1961; Cole, 1970), and delayed recognition (Garfield, 1970; 1980) have been postulated that include 1) information overload from the large amount of information available, 2) modest communication skills of authors, 3) insufficient promotion of ideas, 4) conflict with existing theory and experimental data, 5) the author’s position in the social hierarchy of science, 6) multiple discovery, 7) the management structures of scientific institutions, 8) and the conservative nature of establishments.

The Sleeping Beauty phenomenon, and variants of it, have been extensively studied in different datasets, and some degree of agreement exists that a fraction of the scientific literature exhibits citation kinetics that suggest delayed but eventual recognition of new ideas (Glänzel et al., 2003; Redner, 2005; Braun et al., 2010; Li, 2014). The size of this fraction has received different estimates as well as criteria for defining these estimates (Glänzel and Garfield, 2004; Ke et al., 2015; Li and Ye, 2016; van Raan and Winnink, 2019). Imaginative metaphors have also emerged to describe Sleeping Beauty variants that have been subsequently discussed in terms of their precision and impact (Sugimoto and Mostafa, 2018).

While earlier studies examined small datasets, subsequent ones have considered large samples of the literature, for example, 22 million publications in Ke et al. (2015). In studying the Sleeping Beauty phenomenon, both parameterized and parameter-free approaches have been used (van Raan, 2004; Costas et al., 2010; Li et al., 2014; Ke et al., 2015; Ye and Bornmann, 2018) with partially overlapping results.

While the research cited above has focused on single publications, new ideas also result from combining two previously independent ones. The recognition of such novel ideas can be examined by co-citation analysis (Marshakova-Shaikevich, 1973; Uzzi et al., 2013; Boyack and Klavans, 2014; Wang et al., 2017; Bradley et al., 2020). Co-citation analysis has also been used to identify the so-called “princes” that awaken Sleeping Beauties by (Teixeira et al., 2017; Zong et al., 2018).

Delayed recognition in co-cited article pairs has been briefly explored (Devarakonda et al., 2020) using simplified criteria derived from prior Sleeping Beauty studies on single publications (van Raan, 2004; Ke et al., 2015; van Raan and Winnink, 2019). The authors of this study (Devarakonda et al., 2020), which examined 33.6 million pairs, reported 24 co-cited pairs exhibiting delayed recognition in the 99th percentile of 33.6 million co-citation frequencies, and proposed the term *delayed co-citations* for such cases. While this initial exploration, albeit at scale, only considered reference pairs where each member of a pair was in the 99th percentile of highly cited articles in Scopus, its results suggest that delayed recognition in co-cited pairs is relatively uncommon.

In this article, we examine a much larger dataset, approximately 940 million pairs of articles. We identify co-cited article pairs that exhibit delayed recognition according to criteria derived from the work of van Raan (2004); van Raan and Winnink (2019) and Ke et al. (2015). We also ask whether individual articles found in delayed co-citation pairs can be labeled as Sleeping Beauties.

## 2. Materials and Methods

We have previously described a dataset of 33.6 million cited pairs each belonging to the top 1% of cited articles in the Scopus bibliography (Devarakonda et al., 2020; Figure 2). In the present study, we include all co-cited pairs from references cited by articles published in Scopus in the 11 year period, 1985–1995, not only those drawn from the top 1% of cited articles.

To assemble and analyze a working dataset, we first exported 95,524,693 publication records from Scopus (all citation types) as a citation graph consisting of an edgelist and a nodelist, imported these data into a graph database (Neo4j) treating publications as nodes and citations as edges. After creating indexes to improve performance, we selected all publications of citation type “article” published in the years 1985–1995 (inclusive of both) that had at least five cited references each. In counting references, we only considered references with complete Scopus records. Incomplete references and those with cryptic placeholder identifiers were removed from the dataset. We also filtered rare cases in the data where a publication cites itself, or if the publication date of a cited reference was missing or greater than the publication date of its citing article. Selection of publications with at least five references was performed after curating references.

We used a combination of SQL, Cypher, and *Python* to manage and analyze this volume of data. After initial comparison of SQL vs Cypher, we chose, on the basis of simplicity and performance, to use Cypher queries in Neo4j to generate all pairwise (n 2) combinations of an article’s cited references. We de-duplicated these pairs across all articles to assemble a dataset of ∼940 million pairs (940,357,633 pairs). We then calculated the frequency of co-cited pairs.

For efficiency, we divided the data into batches for parallel processing using the Neo4j 4.0 graph database and the GNU Parallel utility. After tuning experiments on a test set of one million pairs using Neo4j in a Centos 7.5 virtual machine with 128 Gb of RAM and 16 vCPUs in the Microsoft Azure environment, we set batch size to 1,000 pairs and the degree of parallelization to 15 cores. Under these conditions, it took roughly 11 min to compute co-citation frequencies for a batch of 1,000 pairs. We divided these 940 million pairs into nine subsets of around 100 million pairs each and processed them at the rate of approximately 19 h per subset. Our code for parsing and updating Scopus XML data, a PostgreSQL schema for Scopus data, SQL, Cypher, and *Python* scripts used in this study is freely available from a Github repository (Korobskiy et al., 2019).

The simple Cypher query we used to calculating co-citation frequencies of pairs in Neo4j is shown below. The input to the query is a csv file containing two columns of article identifiers with each row representing a co-cited pair.

**Table:** 

UNWIND $input_data AS row
MATCH (a: Publication {*node_id: row.cited_1*})<--(p)-->(b: Publication{*node_id:row.cited_2*})
RETURN row.cited_1 AS cited_1, row.cited_2 AS cited_2, count(p) AS scopus_frequency;

Frequencies thus calculated, were loaded back into PostgreSQL. For kinetic analysis, we selected all pairs with a co-citation frequency >= 100 and calculated the kinetics of citation accumulation from the first possible year of co-citation for each pair through the year 2018, again in Neo4j. Finally, for continuity, we set zero as the frequency for all years between the first possible year of co-citation and the last co-cited year (2018), with missing frequency counts. Minor differences between the data in Devarakonda et al. (2020) are due to more current data in Scopus in our study, and computing kinetic data through 2018 in this study. We compared small samples between the two datasets and confirmed that these minor differences in co-citation frequencies could be bridged by including citations from publications in 2019 and later.

After generating a dataset of 940 million pairs, we applied three relatively conservative conditions to identify cases of delayed co-citation: 1) a minimum peak (annual) co-citation frequency for a pair of at least 20; 2) a minimum total co-citation frequency of at least 100; 3) a requirement both members of a co-cited pair should be published no earlier than 1970. We then identified delayed co-citation cases by setting two more conditions: 1) a minimum sleeping duration of 10 years as measured from the first possible year of co-citation (the more recent publication year of the two articles), 2) during this sleeping period of 10 years or more, the average co-citation frequency should be at most 1 with no more than two co-citations in any one year.

We calculated the Beauty Coefficient using the equation below for 1) a single article as described in detail by Ke et al. (2015), and 2) for co-cited pairs as described in Devarakonda et al. (2020); we treated the first possible year of co-citation equivalently to the year of publication for a single article.

$$B=\overset{t_{m}}{\underset{t=0}{\sum }}\frac{\frac{C_{t_{m}}-C_{0}}{t_{m}}\cdot t+C_{0}-C_{t}}{max\left\{1,C_{t}\right\}}$$

where B is the Beauty Coefficient, *t* is a point in time describing the age of a publication, and $C_{t}$ is the number of citations accrued. at time *t*.

We also calculated the slope between the co-citation frequency of the awakening year and the peak frequency. For single publications, we narrowed the criteria of van Raan and Winnink (2019) to consider only one sleeping period of 10 years or greater; depth of sleep (average citation rate during sleep) of at most 1; an awakening period of 5 years; and an average co-citation frequency during the awakening period (which is defined as awakening citation intensity by van Raan) of at least 5.

We use the term Sleeping Beauty when referring to delayed recognition in individual articles that were identified using prior methodology. For co-citations, we use delayed co-citation or delayed recognition.

## 3. Results and Discussion

**FIGURE 1:**
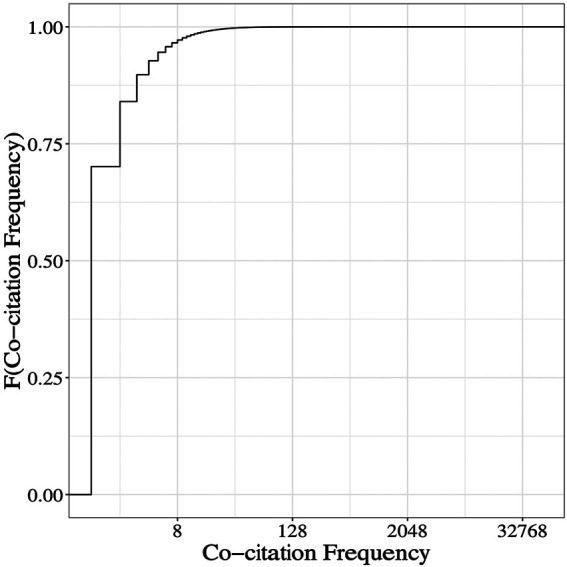
Frequencies of ∼940 million co-cited pairs drawn from Scopus 1985–1995. Pairwise combinations, (n 2), of references from articles indexed in Scopus (1985–1995), were generated as described in Materials and Methods. Total co-citation frequencies for these pairs, ranged from 1 to 52,471 with a median frequency of 1. The empirical cumulative distribution function (ECDF) was calculated from 940,357,633 co-citation frequencies and plotted against co-citation frequencies on a log_2_ scale.

In this study of delayed co-citations, we first examined cited references from 3,433,578 publications in the Scopus database. The criteria for selection of these publications were that they were classified as “article,” that they were published in the period 1985–1995, and they contained at least five cited references each. We generated all possible co-cited pairs for the references in these articles and de-duplicated them across articles. since the same reference pair can occur in more than one article. Then we measured the co-citation frequency of each pair across the entire Scopus database by counting all co-citation events from the first possible year of co-citation onwards through 2018 (Figure 1; Table 1).

**TABLE 1:** Distribution of 940 million Co-citation Frequencies. The count of co-cited pairs in each frequency class as well as the percentage relative to the total number of 940,357,633 is shown. Counts include the lower bound in each class and exclude the upper bound.

*f* interval	Count	Percentage
≦2	790,189,114	84.03
2–4	82,022,893	8.72
4–8	41,772,728	4.44
8–16	17,749,436	1.89
16–32	6,429,234	0.68
32–64	1,704,908	0.18
64–128	385,923	0.041
128–256	81,164	0.0086
256–512	17,150	0.0018
512–1,024	3,777	0.00040
1,024–2,048	948	0.00010
>2,048	358	0.000038

The data in Figure 1 show a highly skewed distribution of co-citation frequencies across a large dataset. Roughly 84% of the pairs have a total co-citation frequency of two or less, and the 99th percentile is 16 although each pair had at least 10 years to accumulate co-citations. Even for a pair of articles from the most recent year in our data, 1995, this frequency of 16 corresponds to less than one co-citation per year on average. Thus, only a small fraction of pairs in these data have co-citation frequencies greater than two per year. One might consider that the reasons advanced for delayed recognition described in the Introduction could also contribute to such modest recognition or even acknowledgment of non-merit.

Beyond a high level understanding of the distribution of co-citation frequencies, however, we are interested in frequently co-cited publications, which are derived from highly cited publications (Small, 1973), and are of interest to the community. Thus, we subset the data using a conservative threshold of 100 for total co-citation frequency along with a peak annual co-citation frequency of at least 20. These criteria are analogous to those proposed by van Raan (2004) and Redner (2005). After applying these two further restrictions, the number of co-cited pairs in consideration was reduced to 51,613 (approximately 0.055% of the total number of pairs).

We applied further conditions to these 51,613 pairs to determine whether they qualified as cases of delayed co-citation: 1) a co-cited pair should have experienced dormancy in citation (a period of “sleep”) for at least 10 years during which it should have received no more than two co-citations per year. This period of dormancy ended in the first year that the pair received more than two co-citations. To be labeled a case of delayed recognition, we also required that the awakening period that follows the sleeping period was characterized by 2) a peak annual co-citation frequency of at least 20. These criteria when collectively applied, identified 1,196 cases of delayed co-citation, whose characteristics are summarized in Table 2. We also note that roughly 18% (223/1,196) pairs were connected by direct citation to each other.

**TABLE 2:** Summary Statistics of 1,196 Delayed Co-citation Pairs. Criteria for selection were a minimum sleeping period of 10 years and a minimum peak of 20 citations in any year. Q1 and Q3 refer to the first and third quartile respectively.

	Total frequency	Sleep duration	Slope	Beauty coefficient*
Min	20.00	10.00	0.21	34.21
Q1	22.00	11.00	1.23	89.40
Median	26.00	14.00	1.700	128.53
Mean	34.06	15.11	2.40	167.63
Q3	36.00	17.00	2.67	190.93
Max	296.00	38.00	38.00	1,678.62

Interestingly, these 1,196 pairs are derived from only 1,267 of a possible 2,392 individual publications indicating that some members of frequently co-cited pairs are found in multiple pairs. This observation is consistent with a pair of articles concerning methods in biochemistry, contributing to over 40,000 different co-cited pairs with frequencies of at least 10 (Devarakonda et al., 2020).

A logical question is whether any of these 1,267 individual publications would exhibit delayed recognition (be classified as Sleeping Beauties). Applying van Raan’s criteria (Section 2), we identify 128 of these 1,267 publications. Interestingly, 27 of the 1,196 delayed co-citation pairs were cases where both members of a delayed co-citation pair would qualify as Sleeping Beauties. Thus, delayed recognition can occur without a requirement that at least one member of a co-cited pair with delayed recognition should have Sleeping Beauty characteristics. These observations also suggest that while high-referencing fields such as biology (Small and Greenlee, 1980) might be advantaged by our selection criteria, the thresholds we set do not entirely exclude other fields. Accordingly, continuing this work with field normalization of co-citation frequencies, to the extent possible, is warranted.

**FIGURE 2:**
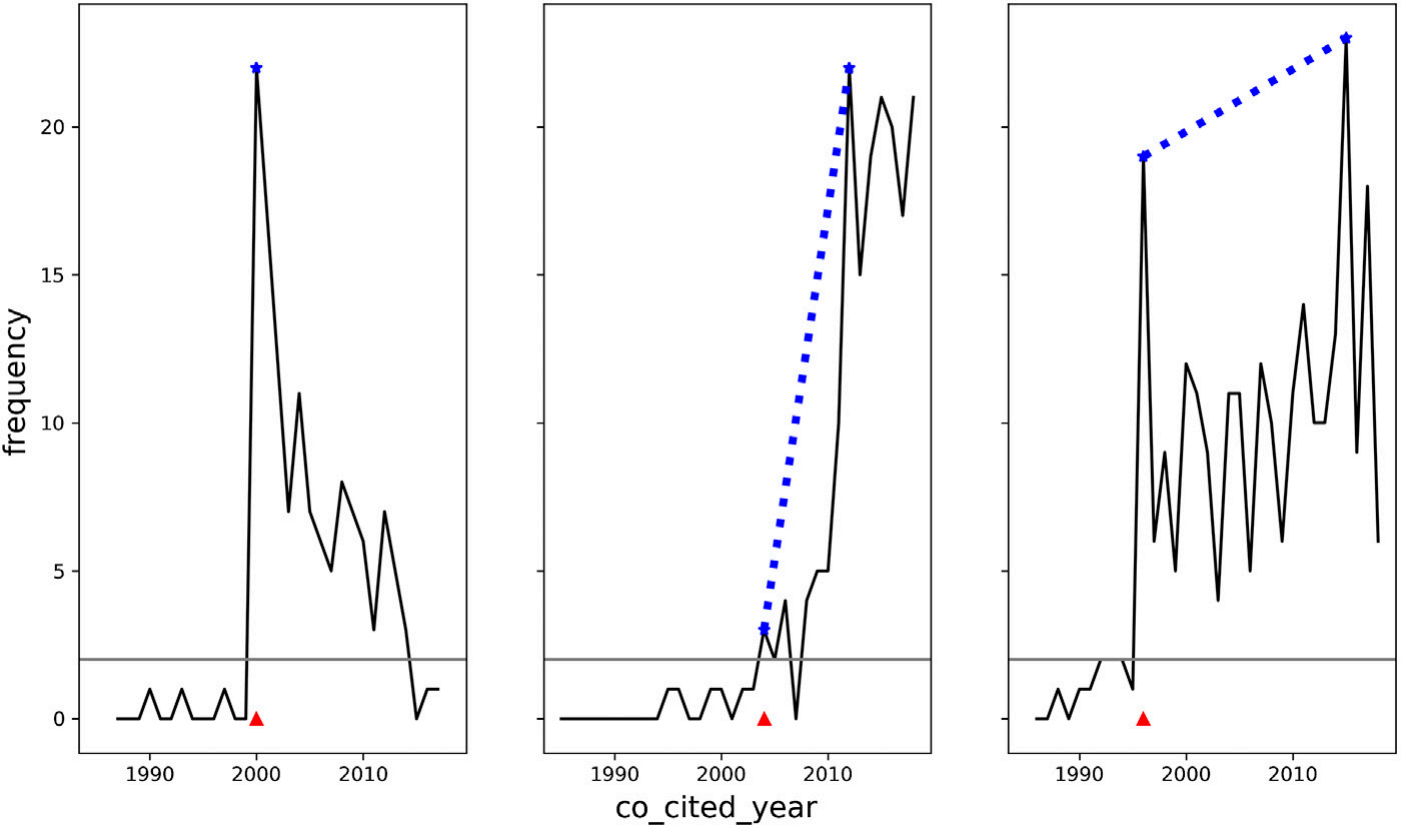
Kinetics of Co-citation Frequencies for Delayed Co-citations. Three sample plots are shown from 1,196 delayed co-citations selected for maximum slope (left panel). mean slope (middle panel), and minimum slope (right panel) of a line connecting the co-citation frequency of the awakening year to the co-citation frequency of the peak year. Total co-citation frequencies for these three plots were 131, 174, and 254, with peaks of 22, 22, and 23, and slopes of NA, 2.38, and 0.21, respectively. The red triangle marks the awakening year and the dotted blue line, the slope. The slope in the left panel is NA since the peak year is the awakening year. The article pairs shown above are 1) Spacetime as a membrane in higher dimensions (Gibbons 1987) and An exotic class of Kaluza-Klein models (Visser, 1985), 2) Formulation of the reaction coordinate (Fukui 1970) and Ab initio effective core potentials for molecular calculations. Potentials for main group elements Na to Bi (Wadt and Hay, 1985), 3) A proposed grading system for arteriovenous malformations (Spetzler, 1986) and arteriovenous malformations of the brain: Natural history in unoperated patients (Crawford et al., 1986).

In contrast to co-citation frequencies for delayed co-citations (Figure 2), which range from 20 to 260; citation counts for the 1,267 publications that contribute to these 1,196 delayed co-citations range from 121 to 190,832 with 72 of these publications having citation counts of greater than 10,000.

However, other co-citation frequencies do exceed the seemingly modest frequencies noted for delayed co-citations. For example, Becke (1993) and Lee et al. (1988), a pair of articles from the field of physical chemistry, have been co-cited over 51,000 times but do not exhibit delayed citation kinetics. It should also be noted that these articles have individually been cited over 70,000 times each. Similarly, 1,357 pairs from the data shown in Figure 2 have co-citation frequencies greater than 1,000.

We observe (Figure 1), that the 90th, 95th, and 99th percentiles of co-citation frequencies in our dataset are 4, 6, and 16 respectively. In comparison. the 90th, 95th, and 99th percentile of citation frequencies of ∼10.7 million publications of type “article” in Scopus, published in the years 1970–1995, are 58, 96, and 254 respectively (roughly ten fold greater). What emerges is that delayed co-citations tend to have frequency profiles that are lower than those of other co-cited pairs, and single publications. This is not unexpected since co-cited frequencies cannot exceed the citation frequencies of the publications in these pairs but it does suggest that seemingly low co-citation frequencies should not be overlooked.

To examine rates of awakening, we also calculated the slope between the co-citation frequency in the first awakening year and the frequency of the peak year and noted a fairly broad range of slopes with a mean of 2.4 (Table 2). The kinetics of co-citation are visualized in Figure 2, for three examples with the maximum slope, the mean slope, and the minimum slope observed.

Of 1,196 delayed co-citations, the slope could not be computed for 10 pairs because the peak year was the year of awakening. This small number of cases, suggest sudden recognition of the concepts represented by these pairs (Table 3. These 10 pairs span the areas of LED technology, cosmology, immunology, psychology, and computational science. One publication from 1985 titled, “An exotic class of Kaluza-Klein models” appears in 3 of 10 pairs and the author himself refers, in 1999, to “renewed interest due to the explosion of activity in the non compact extra dimensions variant of the Kaluza Klein model” (Visser, 1999).

**TABLE 3:** Co-cited pairs with peak frequency in the first year of awakening.

Years	Title
1974	Fundamental energy gap of GaN from photoluminescence excitation spectra
1971	Absorption, reflectance, and luminescence of GaN epitaxial layers
1986	Dimensional reduction caused by a cosmological constant
1985	An exotic class of Kaluza-Klein models
1987	Spacetime as a membrane in higher dimensions
1985	An exotic class of Kaluza-Klein models
1985	An exotic class of Kaluza-Klein models
1985	Do we live inside a domain wall?
1971	Mental rotation of three-dimensional objects
1976	Demonstration of a mental analog of an external rotation
1974	Biologic and clinical significance of cryoglobulins. A report of 86 cases
1980	Mixed cryoglobulinemia: Clinical aspects and long-term follow-up of 40 patients
1977	Imitation of facial and manual gestures by human neonates
1979	Matching behavior in the young infant
1978	Cognitive determinants of fixation location during picture viewing
1979	Framing pictures: The role of knowledge in automatized encoding and memory for gist
1983	Parst: A system of fortran routines for calculating molecular structure parameters (truncated)
1983	On enantiomorph-polarity estimation
1980	Toward a positive theory of consumer choice
1973	On the psychology of prediction

We also examined lesser co-citation frequencies, between 20 and 100, and observed 5,928,815 pairs. After removing pairs with 1) less than 10 years of kinetic data (the difference between publication year and peak year is less than 10 years) 2) a negative Beauty Coefficient, which describes articles whose citations growing linearly with time or with a citation trajectory that is a concave function of time, 3) without at least one peak of frequency 20, then the number reduced to 13,057 pairs. Of these 12,920 had only a single peak of 20 or greater and may be similar to “flash in the pan” citations (Costas et al., 2010; Li, 2014). Given our focus on frequently co-cited pairs, we did not study these further.

An appealing alternative approach for delayed co-citations and Sleeping Beauties is the Beauty Coefficient. We computed the Beauty Coefficient (Materials and Methods) for these 1,196 pairs observing a range of 34.21–1678.62. These data are summarized in Table 2. Given co-citation frequencies being generally lower than citation frequencies, the top 15 Beauty Coefficient values of the 1,196 delayed co-citations range from 712.47 to 1678.62, which appear comparable given lower co-citation frequencies to the top 15 single articles described by Ke et al. (2015), all above 2,000.

Ke and colleagues comment that parameterized approaches in preceding studies have suffered from being somewhat arbitrary. Arbitrariness may not have impeded discovery, for example Redner’s work on the physics literature (Redner, 2005) with its selection threshold of 250 citations. Further, while the Beauty Coefficient is parameter free, the choice of selection threshold is left to the user leaving the door open for arbitrary selection thresholds. We consider this a strength of the measure since it can be used in contextual studies. The approach of van Raan is also intuitive and flexible but does not consider the maximum number of citations received as an important parameter to be tuned. The cases with a sleeping period of ten years, and a citation rate of 5 for the next 5 years, would satisfy requirements for delayed recognition but are perhaps less noteworthy.

**FIGURE 3:**
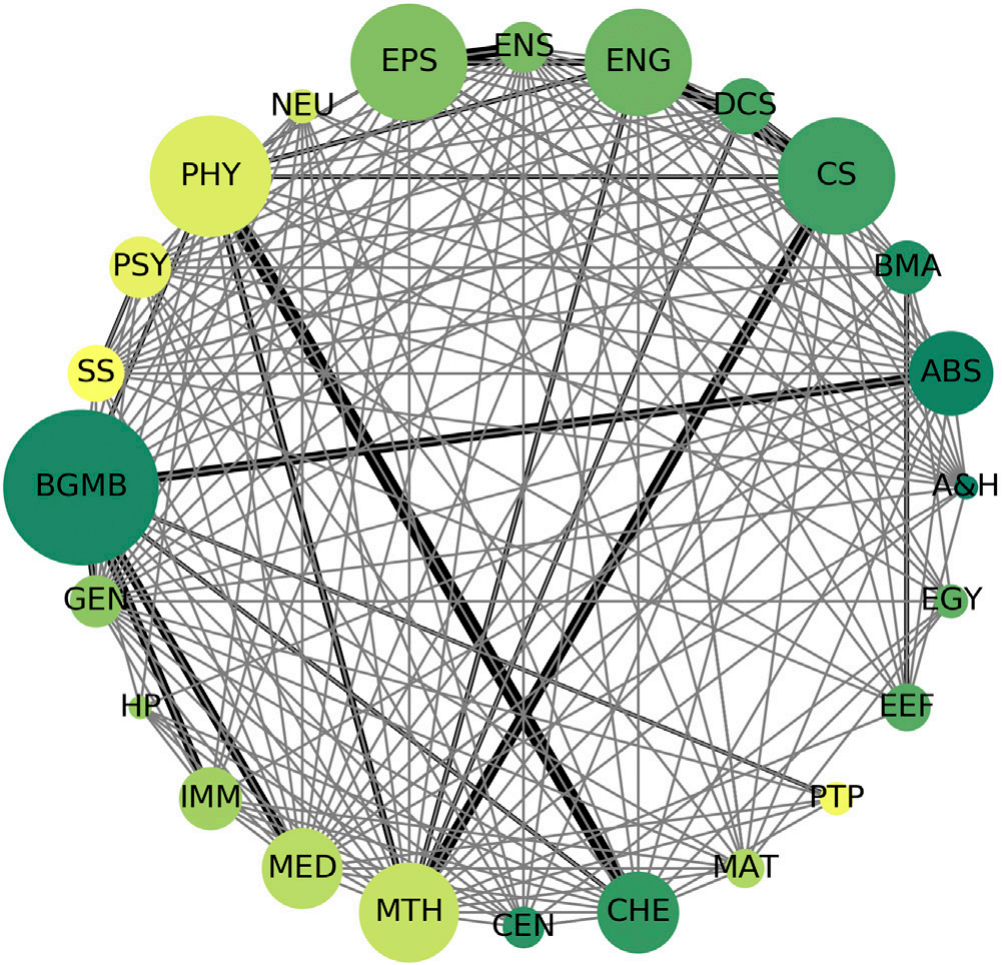
Disciplinary composition of 1,196 Delayed Co-citations. Each node represents a major subject area in the Scopus ASJC classification. Node size is scaled to the number of articles in a given subject area. Edge thickness indicates the number of pairs with one member in each of the two nodes connected by the edge. Major subject areas are abbreviated in the graphic: *BGMB*, Biochemistry, Genetics and Molecular Biology; *SS*, Social Sciences; *PSY*, Psychology; *PHY*, Physics and Astronomy; *NEU*, Neuroscience; *EPS*, Earth and Planetary Sciences; *ENS*, Environmental Science; *ENG*, Engineering; *DCS*, Decision Sciences; *CS*, Computer Science; *BMA*, Business, Management, and Accounting; *ABS*, Agricultural and Biological Sciences; *A&H*, Arts and Humanities; *EGY*, Energy; *EEF*, Economics, Econometrics and Finance; *PTP*, Pharmacology, Toxicology and Pharmaceutics; *MAT*, Material Sciences; *CHE*, Chemistry; *CEN*, Chemical Engineering; *MTH*, Mathematics; *MED*, Medicine; *IMM*, Immunology and Microbiology; *HP*, Health Professions; *GEN*, General.

Finally, to ask which fields these 1,196 delayed co-citations are found in, we mapped them to the All Science Journal Classification (ASJC) maintained by Scopus, which consists of 27 major subject area categories. The data are represented in Figure 3 but should be interpreted in the light of these subject area labels being derived from journals and that an article may have more than one label. Even so, the data suggest that delayed co-citations, as we define them in our dataset are largely drawn from the domain of biochemistry, genetics, and molecular biology followed by physics, computer science, chemistry, and engineering. These observations are slightly different from (Ke et al., 2015; **Figure 4**) with Biochemistry, Genetics, and Molecular Biology dominating in our set but those authors studied single publications from a different data source, and a different time period.

## 4. Conclusion

In a large-scale exploration of the kinetics of co-citation (more than 940 million unique article pairs), we have identified 1,196 cases of delayed co-citation using criteria largely derived from the work of van Raan and Ke. We acknowledge that our selection criteria, while guided by positional statistics and intuitive preference, suffers from some degree of arbitrariness. As with all bibliometric data, coverage and data quality also influence discovery. Thus, we have tried to identify co-cited pairs of higher frequency since the trends in such cases are more likely to be reproducible across other data sources. Relaxing these conditions, will identify additional cases. Our goal was to identify a set of delayed co-cited pairs that can be studied, in the longer term, to understand the reasons for the patterns of citation. This future task will require a greater understanding of the fields in which such delayed co-citations occurred and ideally should be coupled to qualitative techniques. Resolving these observations in a finer-grained manner with respect to kinetics and discipline would also be informative.

## Data Availability

The datasets presented in this article are not readily available because Scopus data has licensing restrictions and cannot be redistributed. The studies are reproducible to persons with a license from Elsevier, the vendor of Scopus. Requests to access the datasets should be directed to netelabs@nete.com
